# Assisting differential clinical diagnosis of cattle diseases using smartphone-based technology in low resource settings: a pilot study

**DOI:** 10.1186/s12917-017-1249-3

**Published:** 2017-11-09

**Authors:** Tariku Jibat Beyene, Amanuel Eshetu, Amina Abdu, Etenesh Wondimu, Ashenafi Feyisa Beyi, Takele Beyene Tufa, Sami Ibrahim, Crawford W. Revie

**Affiliations:** 1 College of Veterinary Medicine and Agriculture, Addis Ababa University, POBox 34, Bishoftu/Debre Zeit, Ethiopia; 2 Business Economics Group, Wageningen University, Hollandseweg 1, 6706 KN Wageningen, The Netherlands; 3 Department of Animal Sciences, University of Florida, Gainesville, FL USA; 4 Cojengo Ltd, Glasgow, UK; 5 Department of Health Management, Atlantic Veterinary College, University of Prince Edward Island, Charlottetown, PEI Canada

**Keywords:** Cattle disease, Differential diagnosis, Ethiopia, Smartphone-based application, Bayesian inference

## Abstract

**Background:**

The recent rise in mobile phone use and increased signal coverage has created opportunities for growth of the *mobile Health* sector in many low resource settings. This pilot study explores the use of a smartphone-based application, *VetAfrica-Ethiopia*, in assisting diagnosis of cattle diseases. We used a modified Delphi protocol to select important diseases and Bayesian algorithms to estimate the related disease probabilities based on various clinical signs being present in Ethiopian cattle.

**Results:**

A total of 928 cases were diagnosed during the study period across three regions of Ethiopia, around 70% of which were covered by diseases included in *VetAfrica-Ethiopia*. Parasitic Gastroenteritis (26%), Blackleg (8.5%), Fasciolosis (8.4%), Pasteurellosis (7.4%), Colibacillosis (6.4%), Lumpy skin disease (5.5%) and CBPP (5.0%) were the most commonly occurring diseases. The highest (84%) and lowest (30%) levels of matching between diagnoses made by student practitioners and *VetAfrica-Ethiopia* were for Babesiosis and Pasteurellosis, respectively. Multiple-variable logistic regression analysis indicated that the putative disease indicated, the practitioner involved, and the level of confidence associated with the prediction made by *VetAfrica-Ethiopia* were major determinants of the likelihood that a diagnostic match would be obtained.

**Conclusions:**

This pilot study demonstrated that the use of such applications can be a valuable means of assisting less experienced animal health professionals in carrying out disease diagnosis which may lead to increased animal productivity through appropriate treatment.

## Background

Agriculture is a source of livelihood for an estimated 2.5 billion people and provides employment for 1.3 billion people globally [1, 2]. In developing countries, such as Ethiopia, livestock contribute around 30% of agriculturally related income [3]. Ethiopia has the largest livestock population in Africa, with cattle representing the largest segment at around 56 million animals [4]. Despite the fact that the economic contribution of livestock and their products account for around 20% of the total gross domestic product [5], 45% of the agricultural gross domestic product [5, 6], and directly contribute to the livelihoods of about 65% of Ethiopian families [7], the country has yet to fulfil its potential in this sector [8]. Numerous factors affect livestock production and productivity of which livestock disease is one of the most important [9]. This factor is exacerbated by lack of access to experienced veterinary services and advice, and consequently the mis-diagnosis and incorrect treatment of endemic cattle diseases.

In remote areas of Ethiopia, where most animal health assistants and community animal health workers practice, there is little access to continued professional development or to quality reference materials to which such practitioners can refer in cases of multiple tentative diagnoses [10]. Furthermore, under field conditions clinical diagnosis of cattle diseases can be complicated by similarity of clinical presentation [11]. So, where multiple similar signs co-occur, decision support tools can facilitate differential diagnosis [12].

Traditionally, the high burden of disease combined with large rural populations and limited infrastructure, has posed significant challenges for those seeking to tackle animal and human health issues. However, the recent rise in mobile phone usage, increased signal coverage and availability of low cost handsets, has created opportunities for growth in the nascent *mobile Health* sector in many low resource countries [13]. Over the past few years, a variety of *mobile-Health* projects have illustrated that mobile technology can be an appropriate vehicle to deliver medical and agricultural knowledge [14, 15] in a flexible and dynamic manner, as well as for the collection of field-based data [16]. The emergence of a number of software start-ups and technology vendors, such as Microsoft (www.microsoft.com/africa/4afrika/), focussing on Africa as an emerging market [17], illustrate the fact that this is a maturing sector with significant potential.

Previous studies carried out in sub-Saharan Africa have demonstrated that the use of low cost and accessible support tools can aid differential diagnosis and significantly improve the performance of animal health workers [18]. A pilot study in Uganda of a paper-based system not only demonstrated its value to the specific task of differential diagnosis in individual animals but also illustrated the utility of information on clinical signs and disease diagnoses in helping address general epidemiological questions related to syndromic surveillance and proportional disease morbidity [18].

However, further research was required if these early successes were to evolve into operational systems; both in terms of validating the robustness of such systems as well as to demonstrate that these novel diagnostic tools can have significant impacts on the health of cattle and livelihoods in rural communities. In this pilot study we document the introduction of a smartphone-based application, *VetAfrica-Ethiopia*, and explore its potential use in the differential diagnosis in cattle disease.

## Methods

### Study area and population

**Fig. 1:**
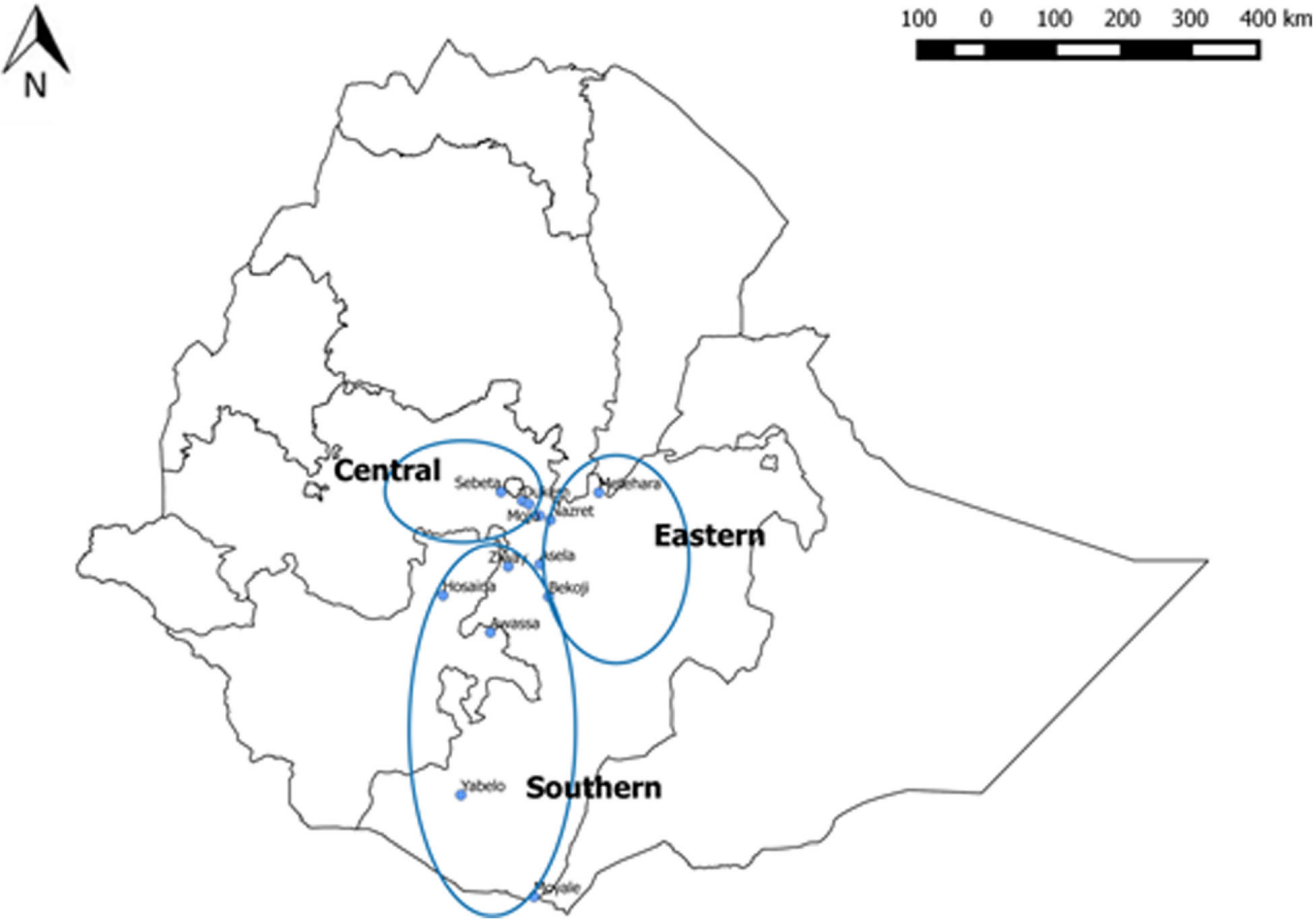
Map of Ethiopia showing the three regions covered by the study

This study was conducted in 13 public veterinary clinics in the central (3 clinics), eastern (5 clinics) and southern (5 clinics) regions of Ethiopia shown on Fig. 1. Fifteen final-year veterinary medicine students from the College of Veterinary Medicine and Agriculture of Addis Ababa University took part in the study. The clinics were selected based on their willingness to provide students supervision, while each student was allocated to a specific veterinary clinic based on the colleges’ assignment for final year field clinical practice (fieldwork and case management) in different regions. Three female students were assigned to the same public veterinary clinic in the town in which the College is based (Bishoftu). The students were given basic training on how to use the smartphone app in clinical case management including how to record cases, connect to Internet and ensure that cases had been updated on the Cloud server, as well as to carry out rudimentary trouble-shooting such as restarting pages and editing data entry errors.

### Disease selection

The initial selection of cattle diseases to be included in the smartphone-app was based on an interaction, using a modified Delphi protocol [19], with 17 experienced veterinarians from the College of Veterinary Medicine and Agriculture of Addis Ababa University in May 2014. Based on this Delphi exercise, 12 diseases were identified as being of particular significance for Ethiopia in terms of economic importance and/or had a high likelihood of providing a challenge in terms of the interpretation of clinical signs. These diseases were: Anthrax, Babesiosis, Blackleg, Contagious Bovine Pleuropneumonia (CBPP), Colibacillosis, Cowderiosis, Fasciolosis, Pasteurllosis, Parasitic Gastroenteritis (PGE), Rabies, Trypanosomosis and Bovine Tuberculosis.

### Development of the *VetAfrica-Ethiopia* smartphone app

Once the diseases had been identified and the related probabilities of various clinical signs determined using simple Naïve Bayes estimation, an application was developed for a smartphone by the commercial partners in the project, Cojengo Ltd. In collaboration with one of the current authors (CWR), Cojengo had in 2014 developed the *VetAfrica* app for use in Uganda and Kenya. A revised version of the app, *VetAfrica-Ethiopia* (VAE), which in addition to being targeted towards appropriate diseases for use in Ethiopia also applied a Naïve Bayes classier to predict disease class, was distributed to the 15 student practitioners on low-cost smartphones.

As part of each student’s regular fieldwork they assessed the disease status of cattle which were brought to the veterinary clinic in their district. A complete clinical examination was conducted on sick animals by the student practitioners under the supervision of the veterinarians on duty in the respective clinics. The basic data recorded in the *VetAfrica-Ethiopia* app included: date of examination; location of the case (village); breed, sex and age of the animal; the presenting clinical signs (or absence of same) were recorded and a tentative diagnosis was made by the student practitioner. Based on those signs that were reported to be either present or absent, the *VetAfrica-Ethiopia* app estimates the predicted likelihood for a range of tentative diagnoses in descending order of probability. The student practitioner therefore proposed a diagnosis using the clinical signs and any other relevant case history, while the app gave a range of tentative diagnoses with associated likelihoods. All of these data were then uploaded to a *Cloud*-based server (Microsoft Azure) for further analysis.

### Estimation of proportional morbidity

The relative frequencies of a range of diseases from the total number of animals presenting at the public veterinary clinics during the study period, as diagnosed by the student practitioners, were computed to provide an estimate of proportional morbidity.

### Evaluation of *VetAfrica-Ethiopia*

An evaluation of the level of agreement between the student practitioner’s diagnoses and the prediction(s) of *VetAfrica-Ethiopia* was carried out. First we constructed a simple misclassification matrix to identify the degree of match for each disease. One problem with this simplistic approach to defining a ‘match’ is that it is sensitive to small changes in the probabilistic estimates associated with the diagnostic outcomes from the *VetAfrica-Ethiopia* app. For example, imagine a case where the student provided a diagnosis of “Blackleg” and *VetAfrica-Ethiopia* provided the following estimates: (Blackleg = 0.42) and (CBPP = 0.39). Strictly speaking this is a “match”. However, *VetAfrica-Ethiopia* has also indicated that CBPP is highly likely and we may just have been ‘lucky’ that Blackleg was shown as 3% more likely. Conversely, what if we had obtained these results in a setting where the student indicated that her diagnosis was “CBPP”; would we not feel that the *VetAfrica-Ethiopia* app was ‘almost’ right? To deal with these situations we decided to label any case where the second most likely diagnosis suggested by *VetAfrica-Ethiopia* was within 20% of the likelihood value of its primary diagnosis, and where one of these two matched the student’s diagnosis, to be a “marginal” case. Thus in the example given above, only when the second diagnosis was less than 0.336 (i.e. 20% lower than 0.42) would we treat Blackleg as a non-marginal case. Clearly in the case where the student stated that the diagnosis was, for example, “PGE” there is no need to consider the ‘marginal’ condition as both of the leading diagnoses proposed by *VetAfrica-Ethiopia* are incorrect. In the analysis we assess the impact of taking this approach to ‘marginal’ cases as well as the value of using the remaining ‘clear-cut’ cases in the regression modelling.

Univariate and multiple-variable logistic regression models were used to explore those variables which could predict the likelihood of there being a ‘match’ between the diagnosis made by the student practitioner and the diagnosis predicted by the *VetAfrica-Ethiopia* app for all non-marginal cases. A range of variables were screened using a univariate approach to look for candidates that should be preserved in the model; these included: the breed, age, and sex of the animals; the total number of clinical signs provided to *VetAfrica-Ethiopia*; a variable which reflected whether or not the diagnosis under consideration had been included in the *VetAfrica-Ethiopia* app; the diagnosis arrived at by *VetAfrica-Ethiopia*; Town (representing the individual student practitioners); and VAE_Max (the score given by *VetAfrica-Ethiopia* to the most likely diagnosis according to the results of its inference calculations, which was a proxy for the level of certainty the algorithm had in that particular diagnostic outcome). Any variable that had a *p*-value of less than 0.20 was considered for inclusion for the final model. A multiple-variable logistic model was then constructed.

## Results

### Characteristics of cases reported by student practitioners

The student practitioners reported on a total of 928 cattle cases that visited the veterinary clinics in the three regions. The breakdown of these cases by breed, sex and age group is shown in Table 1. The table indicates that a relatively higher number of animals were examined in the Southern region (around 40% of the total, with 30% in each of the other two regions). There were significantly more male animals examined (*p* = 0.02) in the Central and East regions, and the majority of cattle belonged to the oldest age category (over 24-months), though the Southern region reported significantly higher proportions (*p* < 0.01) of younger animals. In all regions, over 80% of the cattle presenting were local (zebu) breeds, with limited numbers of exotic and cross-bred animals. There are of course some important associations that are not captured in Table 1. For example, while there were significantly more male animals in total (57% versus 43% female), these proportions were significantly altered for the case of cross-bred (65% female to 35% male) and particularly exotic (79% female to 21% male) animals. This is due to the fact that the imported or cross-bred animals tend to be dairy cattle.

**Table 1:** Breakdown of all cases recorded during the study (*N* = 928) by region and in terms of proportions across key variables within these regions

	Central	Eastern	Southern
Total cases	282	279	367
by Sex			
Female	37.6%	41.2%	48.2%
Male	62.4%	58.8%	51.8%
by Breed			
Cross	3.5%	6.5%	7.9%
Exotic	4.6%	11.8%	4.1%
Local	91.8%	81.7%	88.0%
by Age Group			
0-6 months	1.8%	7.5%	13.1%
7-12 months	2.8%	5.7%	13.4%
13-24 months	11.0%	11.8%	10.9%
> 24 months	84.4%	74.9%	62.7%

### Proportional morbidity

The proportional morbidity (i.e. the relative frequency of each disease from the total of the 928 cases visiting the veterinary clinics during the study period) based on the diagnoses reported by the student practitioners is given in Table 2. In total just over 70 different diseases were diagnosed over the study period. All diseases that had 3 or fewer cases in the study, a total of around 45 separate diseases, are collected into a single category under the label “Other diseases” in Table 2; with the exception of Anthrax for which just a single case was recorded, but this disease has been left in the table as it was included in the *VetAfrica-Ethiopia* app. Around 70% of the cases were associated with a disease that had been identified as important by local veterinary experts and had been included in *VetAfrica-Ethiopia*.

**Table 2:** Summary of proportional morbidity by disease across all cases (N = 928), including an indication as to which diseases were covered by the *VetAfrica-Ethiopia* app

Disease	Proportion	In *VAE*?
PGE	25.8%	Yes
Blackleg	8.5%	Yes
Fasciolosis	8.4%	Yes
Pasteurellosis	7.4%	Yes
Colibacillosis	6.4%	Yes
Lumpy Skin Disease (LSD)	5.5%	No
CBPP	5.0%	Yes
Babesiosis	2.7%	Yes
Lungworm	2.6%	No
Foot and Mouth Disease (FMD)	2.3%	No
Trypanosomiasis	2.2%	Yes
Salmonellosis	1.6%	No
Coccidiosis	1.6%	No
Tuberculosis	1.6%	Yes
Paratuberculosis	1.5%	No
Mastitis	1.4%	No
Actinobacillosis	1.4%	No
Actinomycosis	1.4%	No
Cowdriosis	1.2%	Yes
Pneumonia	0.9%	No
Tick Infestation	0.8%	No
Demodecosis	0.6%	No
Dermatophilosis	0.5%	No
Dermatophytosis	0.5%	No
Rabies	0.5%	Yes
Vesicular Stomatitis	0.5%	No
Anthrax	0.1%	Yes
Other diseases	7.1%	No

The most common diagnosis, by some margin, was Parasitic Gastrointestinal (PGE) disease with almost 26% of the cases. Other relatively commonly occurring diseases, included: Blackleg (8.5%), Fasciolosis (8.4%), Pasteurellosis (7.4%), Colibacillosis (6.4%), Lumpy skin disease (5.5%) and CBPP (5.0%). Of the top 11 diseases, in terms of proportional morbidity, only Lumpy Skin Disease (LSD), Lungworm and Foot and Mouth Disease (FMD) had not been included in *VetAfrica-Ethiopia*. However, some diseases such as Rabies and Anthrax which were listed as important, perhaps due to their status as notifiable diseases, were found to have proportional morbidities of only 0.5% and 0.1% respectively.

### Comparison of diagnoses made by practitioners and *VetAfrica-Ethiopia*

We used a misclassification matrix to explore the level of matching between the diagnosis provided by the student practitioner and that predicted by the *VetAfrica-Ethiopia* app based simply on the highest probability score. This helps to identify those diseases having a high level of discrepancy as shown in Table 3. Accordingly, considering all 928 cases, Babesiosis was the disease with the highest level of matching; of the 25 cases diagnosed by the student practitioner, 21 were also predicted by the VAE app (84%) with 2 mismatches in each of Fasciolosis and ‘Other’ disease. Colibacillosis (81%) was the disease with the next highest level of match., In contrast, relatively low levels of matching were observed for Trypanosomiasis (45%) and Pasteurellosis (30%). While providing an outcome of “other” was a valid option from *VetAfrica-Ethiopia*, of the 281 cases for which the student practitioners made this choice, the app only suggested this to be most likely in 118 (42%) of these cases.

**Table 3:**
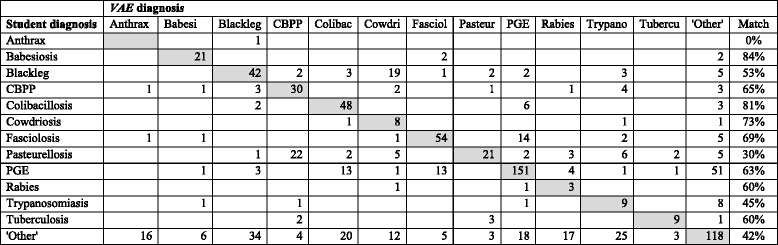
Misclassification matrix for all 928 cases, with student practitioner’s diagnosis and *VetAfrica-Ethiopia* app prediction shown in vertical columns and horizontal rows respectively. Those where the two diagnoses are in agreement are shown in the shaded main diagonal

There were 89 cases for which the probability score predicted by the *VetAfrica-Ethiopia* app for a second diagnostic outcome was less than 20% lower than the probability score of the highest scoring diagnosis; where at least one of these diagnoses matched that proposed by the student practitioner. As can be seen from Table 4, around 67% of these “marginal” cases would have initially been deemed to represent a ‘match’ (i.e. the highest scoring diagnosis suggested by *VetAfrica-Ethiopia* was the same as that of the practitioner). In just under 33% of these cases we would initially have deemed there to be ‘no match’ as the *VetAfrica-Ethiopia* app only had the practitioner’s putative diagnosis ranking as a ‘close second’. It is thus obvious that discounting these marginal cases in no way inflated the apparent performance of the *VetAfrica-Ethiopia* app, if anything it was likely to slightly under-state the level of agreement. However, in seeking to better understand the factors associated with obtaining the same outcomes between practitioners and the app, it seemed wise to exclude these 89 intrinsically ‘confusing’ cases.

**Table 4:** Distribution of ‘marginal’ cases by disease (*n* = 89)

	Initially categorised as a match?	
Disease	No	Yes
Babesiosis	–	2
Blackleg	1	2
CBPP	3	10
Colibacillosis	–	2
Fasciolosis	5	1
Pasteurellosis	4	9
PGE	11	11
Trypanosomiasis	–	1
Tuberculosis	–	1
Other diseases	5	21
Total	29	60

**Fig. 2:**
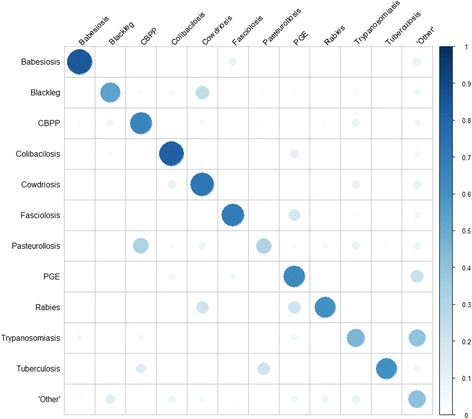
Level of agreement for the non-marginal cases (*N* = 838) by disease in terms of diagnoses made by student practitioner (vertical) and the *VetAfrica-Ethiopia* app (horizontal) diagnoses. Size of circle and intensity of shading represent level of agreement

When considering the 839 (non-marginal) cases that remained, there were some diseases for which the level of match increased and others for which it decreased; for example the level of matching for Babesiosis was 90% (up from 84%) while for Colibacillosis it was 70% (down from 81%). However, the graphical summary in Fig. 2 illustrates that the broad level of agreement across these cases was in line with that seen when considering all 928 animals. (There are in fact only 838 cases summarised in Fig. 2 and in the regression analyses that follow, as the single case of Anthrax in the study has also been excluded.)

### Determinants of diagnosis matching between practitioners and *VetAfrica-Ethiopia*

Univariate logistic analyses were run, using the non-marginal 838 cases, to assess the potential of the available variables to predict diagnostic matching. This demonstrated no significant association (even at the *p* < 0.20 level) for the variables Age and Sex of the animal, or for the total number of clinical signs noted (S_Count). However, the Breed, Region, Town, User_Diag, In_VAE and VAE_Max variables were all found to be significant (p < 0.20) candidate predictors of a match between the student practitioner and the diagnosis made by the *VetAfrica-Ethiopia* app (Table 5).

**Table 5:** Significance of each predictor in a simple univariate logistic model to predict the likelihood of a match (*N* = 838)

Variable Name	Explanation	*P*-value
Age	Age of animal	0.43
Sex	Sex of animal	0.86
Breed	Breed of animal	0.07
S_Count	Number of signs provided for this case	0.72
Region	Region of the country from which case came	0.00
Town	Town within which the student practitioner was working	0.00
User_Diag	The diagnosis provided by the student practitioner	0.00
In_VAE	Was diagnosis listed as a possible outcome within the VAE app?	0.00
VAE_Max	The actual maximum probability score associated with the diagnosis predicted to be the most likely match by the VAE app	0.00

The candidate variables were included in a multi-variable logistic model, with the exception of Region, as each Town (where a student practitioner was based) was situated in a specific geographical region and therefore Region could not be included along with Town. It was also found the Breed, and In_VAE were no longer significant, with the variability in the latter variable being mostly captured by the diagnosis being suggested (User_Diag). This resulted in a final model as summarised in Table 6.

**Table 6:** Summary of multivariable logistic model output for variables predicting a match between the diagnosis provided by the student practitioner and the VAE app (N = 838)

Matched	Coef.	Std. Err.	z	P > |z|	95% Confidence interval	
*User_Diag*						
Blackleg	−1.48	0.63	−2.34	0.02	−2.72	−0.24
CBPP	−1.02	0.70	−1.47	0.14	−2.39	0.34
Colibacillosis	−0.37	0.68	−0.54	0.59	−1.69	0.96
Cowdriosis	−0.76	0.92	−0.83	0.41	−2.56	1.04
Fasciolosis	−0.53	0.65	−0.81	0.42	−1.80	0.75
PGE	−0.95	0.60	−1.57	0.12	−2.13	0.23
Pasteurellosis	−3.26	0.69	−4.72	0.00	−4.61	−1.90
Rabies	−0.50	1.14	−0.44	0.66	−2.74	1.73
Trypanosomiasis	−1.52	0.75	−2.02	0.04	−2.99	−0.05
Tuberculosis	−1.02	0.83	−1.23	0.22	−2.64	0.60
Other	−2.05	0.60	−3.43	0.00	−3.23	−0.88
*VAE_Max*	1.31	0.49	2.69	0.01	0.35	2.26
*Town*						
HOS	−1.73	0.48	−3.58	0.00	−2.68	−0.78
ASS	−0.42	0.55	−0.77	0.44	−1.49	0.65
BI1	−0.80	0.48	−1.69	0.09	−1.73	0.13
BI2	−1.18	0.48	−2.48	0.01	−2.12	−0.25
BI3	−2.80	0.56	−5.00	0.00	−3.90	−1.70
BOK	−0.25	0.46	−0.55	0.58	−1.15	0.65
DUK	−2.72	0.54	−5.03	0.00	−3.78	−1.66
HWS	−0.97	0.53	−1.84	0.07	−2.00	0.07
MAT	−2.01	0.56	−3.56	0.00	−3.12	−0.90
MOD	−1.34	0.50	−2.65	0.01	−2.33	−0.35
MOY	−1.59	0.48	−3.34	0.00	−2.52	−0.66
SEB	−1.03	0.50	−2.04	0.04	−2.02	−0.04
YAB	−1.86	0.49	−3.83	0.00	−2.82	−0.91
ZIW	−1.77	0.44	−4.08	0.00	−2.63	−0.92
_cons	2.10	0.80	2.62	0.01	0.53	3.66

LR chi^2^(26) = 204; Log likelihood = −475

Prob > chi^2^ = 0.00; Pseudo *R* ^2^ = 0.18

**Fig. 3:**
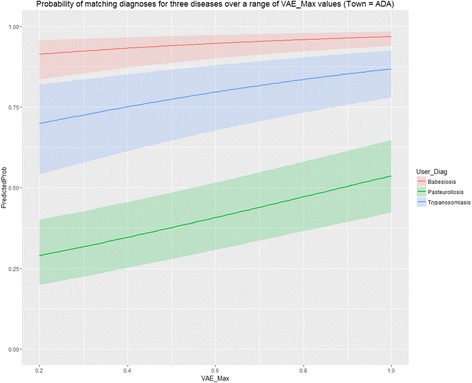
Curves indicating the predicted probability (with 95% CI) of a match for a variety of values of VAE_Max by the algorithm within the VetAfrica-Ethiopia app

As can be seen from Table 6, the likelihood of a match was significantly associated with the disease diagnosed; with Babesiosis (the diagnosis with the highest likelihood of a match) acting as the reference category. There were four disease options (including the catch-all ‘Other’ category) for which the likelihood of gaining a match were significantly lower. The value of VAE_Max was also noted as being a highly significant contributor to the likelihood of obtaining a matched outcome. The impact of this variable is illustrated by the lowess curves shown in Fig. 3, which indicate that as the value of the score associated with the most likely diagnosis (VAE_Max) increases, the likelihood of a match also increases, but with varying slope across diagnoses. For instance, the likelihood of matching does not vary a great deal for the case of Babesiosis (light red), while it does so in the case of Pasteurellosis (light green). The outputs in Fig. 3 are based on a scenario that assumes the student practitioner under consideration was from ADA, the best performing Town in terms of gaining a match. The output in Table 6 indicates that Town (i.e. the student involved) was significantly associated with the chance of a match; with 10 students have significantly less likelihood of obtaining a match than was the case for ADA (the referent student/Town). The full model, including the variables Diagnosis, Town and VAE_Max resulted in an AUC value of 0.77.

## Discussion

In this pilot study we compared the level of match between the *VetAfrica-Ethiopia* app and a set of student practitioners’ diagnoses. It was not possible to confirm cases by laboratory investigation as many clinics were far from suitable laboratories and such investigation would have been highly resource intensive. In the absence of a definitive identification of the disease causing agent, which is assumed to be the gold standard in many cases [20], we still wished to explore the likelihood that the app had made the ‘correct’ diagnosis. Strictly we did not make the assumption that the student practitioners had always suggested the correct diagnosis, but rather assessed how often the student practitioner’s diagnosis and that provided by the *VetAfrica-Ethiopia* app were in agreement. To reduce potential bias in situations with a high degree of uncertainty, we introduced the concept of marginal cases.

It must be acknowledged that this was a pilot study and that as such the results should only be extrapolated with caution. For example, due to financial and logistic constraints we chose to use final year veterinary medicine students to evaluate the use of the *VetAfrica-Ethiopia* app in the field. We are aware that collecting data from students during their clinical rotation is not the same as observing an operational veterinarian in the field. In addition, in many such rural settings diagnostic assistance is provided by animal health technicians or community animal health workers; as such it would be valuable to assess the utility of this type of tool for those communities. There was some facility to capture more general case information within the app, including information of treatment options and notes as to whether samples were collected for laboratory analyses. However, these aspects of *VetAfrica-Ethiopia* functionality were not evaluated within this study. As such a complete consideration of how this type of technology might impact on the full spectrum issues involved in cattle disease management is only partially informed by this work. However, we do believe that the more limited goal set out here, that of assessing diagnostic capability, can be meaningfully summarised.

Misclassification analyses of the 12 identified diseases revealed that the highest level of matching occurred for the disease Babesiosis followed by Colibacillosis, while the lowest level of accuracy was seen in the case of Pasteurellosis. This may be due to the fact that diseases with the highest level of matching such as Babesiosis and Colibacillosis are less challenging in terms of the interpretation of clinical signs and tend to be well characterised. However, diseases with low levels of matching such as Pasteurellosis and Fasciolosis tend to have less clearly distinguished clinical signs or have similarities in terms of their clinical presentation to other diseases [21].

In this study we also found that the likelihood of a diagnostic match between the student practitioner and *VetAfrica-Ethiopia* depended on the particular student making the diagnosis. This could reflect differing degrees of diagnostic competence between students or their ability to use the mobile phone application. In addition each student was under the supervision of a more experienced veterinarian in the clinic to which they were assigned, and some of the student-to-student variation could be due to a differential level of consultation with these supervisors. As their assignment to the various clinics was not random, bias may also have been introduced due to location-specific factors such as variable local mentoring or differential exposure to disease. In addition, the score given by *VetAfrica-Ethiopia* to the most likely diagnosis strongly influenced the chance of there being a match. This suggested that in future versions of the app, some minimum acceptable threshold of score should be set below which the app would state that the diagnosis was “inconclusive” or required additional clinical data, rather than providing putative diagnoses with low likelihoods.

As noted above, a limitation of this study was the inability to confirm the disease in each case based on a laboratory diagnosis. However, as we were attempting to cover a wide geographic area, it was not logistically feasible to access appropriate laboratories. Even if this had been feasible there would likely have been significant uncertainties associated with the diagnostic sensitivity and specificity of results for a number of these diseases [22]. In addition, if we assume a ‘null’ model, i.e. that cases fall on the leading ‘matching’ diagonal merely by chance, our expectation of obtaining a match would be just under 8%; as reported, the actual level of matching observed was more than 7 times this level. However, to aid with more complete validation in future, the authors have arranged workshops with veterinary experts from which one of the key outputs will be a set of ‘control’ cases with defined outcomes that will be available to be used to assess the performance of revised editions of *VetAfrica*.

The 12 cattle diseases of particular significance in terms of trade and economic importance or that were challenging in terms of the interpretation of clinical signs, as identified by experts during the Delphi exercise, were also found to be among the top listed diseases targeted for control by the veterinary services of Ethiopia [23]. The clinical signs chosen for inclusion in the app included those shared among many of the diseases [21]. The assignment of final year veterinary student practitioners to different veterinary clinics during their clinical rotation provided an opportunity to test this relatively low cost technology in a wider agro-ecology with a diverse distribution of livestock diseases. This demonstrated the potential for wider dissemination to veterinary professionals in remote areas. While all of the diseases selected by the experts were of some significance for Ethiopian cattle they are obviously not all equally prevalent. This was a simplifying assumption of this initial instantiation of *VetAfrica-Ethiopia* which may have affected the effectiveness of the diagnostic algorithm. This decision was made due to the absence of detailed disease prevalence estimates in cattle within the areas in which the app was tested. However, the algorithm has been set up to work equally well with any matrix of prior prevalence values as such those estimated as part of the proportional morbidity calculations made here could immediately be used, as could any other data- or model- driven estimates of more accurate priors for disease prevalence.

As far as signs, rather than disease distribution, are concerned the Naïve Bayes approach makes the assumption that each sign is independent with respect to its association with the diseases under consideration. However, the Bayesian Belief Network (BBN) that underlies the algorithmic approach here [24, 25] allows this assumption to be relaxed while maintaining mathematical integrity. Their main challenge, and the reason that the simpler Naïve Bayes assumptions were adopted here in the first instance, lies in estimating the non-independent (conditional) probabilities – which tend to grow in a combinatoric fashion. However, a range of data-driven learning algorithms such as tree-augmented Naïve Bayes (TAN) are available to help made such challenges tractable [26].

The student practitioners were given no instruction to select specific types of case so we assume that the breakdown is broadly reflective of the cases brought to public veterinary clinics seeking diagnostic and treatment services. Male cattle were more commonly seen in these veterinary clinic visits, perhaps reflecting the higher perceived value of males in terms of draft power and market sales value [27]. We found the large majority of the cattle presenting to be of local breed and that over two thirds of the animals were in the over 24-month category. These findings are in line with reports from the Central Statistical Authority of Ethiopia stating about 98.7% of cattle kept in Ethiopia are of indigenous breed and 65.6% are over 3 years of age [4]. Indeed the fact that the proportions of exotic and cross-bred cattle present in our study was well over the 1.3% that might be expected from the census data, may indicate the higher value of such cattle to the farmer and/or their higher level of susceptibility to endemic disease.

The intentions in quantifying proportional morbidity in this study were twofold: to characterise the distribution of disease among the animals examined and to assess the proportion of cases that were covered by those diseases included in the *VetAfrica-Ethiopia* app. The 12 diseases covered by *VetAfrica-Ethiopia* captured around 70% of the putative diagnoses made by the practitioners for cases they attended throughout the study period. The diseases most commonly diagnosed by practitioners were helminthiases: parasitic gastroenteritis (PGE) and bacterial diseases: Blackleg, Pasteurellosis and Colibacillosis. Lumpy skin disease, Lungworm and FMD were found to be diseases that presented relatively more frequently than some which were initially included in *VetAfrica-Ethiopia*, such as Rabies and Anthrax. While these cases were not confirmed by laboratory tests, the disease profile was consistent with the endemic diseases reported to the national veterinary services as published by the Ministry of Agriculture in the animal health year book [23]. They are also reflected in the range of veterinary drugs sold in veterinary drug shops in Ethiopia as authorised by the drug administration and control authority of Ethiopia [28]. However, as the reports included in the current study were based on cattle cases presented to public veterinary clinics they might not be entirely in line with the true proportional morbidity of disease occurrence. It has been reported elsewhere that some cases will be taken to traditional healers, rarely they may be seen by private veterinarians, while some will be left untreated [29–31]. The *VetAfrica-Ethiopia* developers, with inputs from veterinary experts, have now added the three most commonly occurring diseases not included in the app (LSD, lungworm and FMD) so that a set of 15 specific diseases have likelihood estimate values, which would cover just over 80% of the cases seen in this pilot study. One great advantage of a Cloud-based system where cases are available in near real-time is that an alert could easily be set up to let data administrators know when a given disease (currently not included in the app) had been recorded more than some minimum threshold of cases in a given time period. This could then initiate the required work with veterinary experts to explore the inclusion of this additional disease into the app.

The recent increase in availability of low cost mobile handsets and network coverage has created opportunities for applications in the health sector, especially in low resource settings [32, 33]. However, to date these have been predominantly focused in the domain of human health with only limited application to the challenges of diseases in animal populations [34]. Although such smartphone-based applications have several advantages in resource-limited settings, there are also limitations. These include the non-comprehensiveness of the disease list that we constructed at the beginning of the study. In this regard, three additional diseases which accounted for almost 10% of the total cases have been included in a revised version of the app. The diagnostic prediction of the app was also found to have limited accuracy for some diseases. To address this, the Bayesian learning aspect of the diagnostic algorithm supports ongoing modifications in sign-disease weightings. In addition, the fact that the score associated with the most likely diagnosis in VAE strongly influenced the likelihood of practitioner agreement, led to the adoption of a ‘minimum acceptable likelihood threshold’ below which no diagnosis will be suggested by VAE. Despite increased telecoms coverage in Ethiopia in recent years, the technology is still not entirely reliable and there were some instance where delays were experienced in uploading data to the Cloud. This was also in part due to some technical limitations of the Windows Mobile platform used during the study; to help address these, the revised version of VAE is available on Android devices which provides a more seamless interaction between off-line and on-line data access.

This pilot study has demonstrated the potential use of a smartphone-based application for animal disease diagnosis. Correctly diagnosing cattle diseases is known to be a key constraint on animal production efficiency in developing countries. To the authors’ knowledge this is the first attempt to evaluate such an approach in a resource-limited setting. It also seems likely that such an approach would have great potential in other constrained sectors of veterinary service provision such as disease surveillance.

## Conclusions

In conclusion, we have demonstrated that smartphone-based applications can be used by animal health professionals and have several advantages in resource-limited settings. In this pilot study we evaluated the performance of the *VetAfrica-Ethiopia* smartphone-based application based on the level of match between student practitioners’ diagnoses and the app’s predictions. The main findings of this study indicated that an acceptable overall level of matching could be achieved and that the major determinants of such matching were the disease being diagnosed, the diagnostic ability of the student practitioner and the level of certainty the *VetAfrica-Ethiopia* app assigned to the most likely diagnosis. It was shown that the higher this predicted likelihood, the more likely there would be a matching diagnosis, which led to suggestions for design changes in the way that outputs from the algorithm were presented to the user. However, these likelihood values did vary according to disease and it should be noted that some important cattle diseases are currently not captured by the app. In addition the results from a pilot study involving 15 final year veterinary students should be treated with some caution, when drawing broader implication regarding wide-scale adoption. We have begun to explore the potential of smartphone applications such as *VetAfrica-Ethiopia* in providing assistance to less experienced animal health professionals. Further research, involving more definitive case outcomes, is required to fully access improvements in disease diagnosis and the provision of the most appropriate treatment advice; ultimately leading to an increase in animal productivity. We have also illustrated that the careful evaluation of such approaches can lead to better and ultimately more sustainable solutions.
